# Resistin Associated With Cytokines and Endothelial Cell Adhesion Molecules Is Related to Worse Outcome in COVID-19

**DOI:** 10.3389/fimmu.2022.830061

**Published:** 2022-06-16

**Authors:** Takeshi Ebihara, Hisatake Matsumoto, Tsunehiro Matsubara, Yuki Togami, Shunichiro Nakao, Hiroshi Matsuura, Shinya Onishi, Takashi Kojima, Fuminori Sugihara, Daisuke Okuzaki, Haruhiko Hirata, Hitoshi Yamamura, Hiroshi Ogura

**Affiliations:** ^1^ Department of Traumatology and Acute Critical Medicine, Osaka University Graduate School of Medicine, Suita, Japan; ^2^ Osaka Prefectural Nakakawachi Emergency and Critical Care Center, Higashiosaka, Japan; ^3^ Laboratory for Clinical Investigation, Osaka University Hospital, Suita, Japan; ^4^ Core Instrumentation Facility, Immunology Frontier Research Center and Research Institute for Microbial Diseases, Osaka University, Osaka, Japan; ^5^ Genome Information Research Center, Research Institute for Microbial Diseases, Osaka University, Osaka, Japan; ^6^ Department of Respiratory Medicine and Clinical Immunology, Osaka University Graduate School of Medicine, Suita, Japan

**Keywords:** resistin, COVID-19, cytokines, endothelial damage marker, cytokine network

## Abstract

**Introduction:**

Resistin is reported to form a cytokine network and cause endothelial damage. The pathogenesis of coronavirus disease 2019 (COVID-19) remains unknown, but the association between cytokine storm and endothelial damage is crucial. This study aimed to evaluate resistin in COVID-19 pathogenesis compared with sepsis.

**Materials and Methods:**

First, we evaluated the association of plasma resistin levels and disease severity and clinical outcome in two large cohorts: a publicly available cohort including 306 COVID-19 patients in the United States (MGH cohort) and our original cohort including only intubated 113 patients in Japan (Osaka cohort 1). Second, to understand pathogenesis, we evaluate resistin, cytokines and endothelial cell adhesion molecules in COVID-19 compared with sepsis. Blood samples were collected from 62 ICU-treated COVID-19 patients and 38 sepsis patients on day 1 (day of ICU admission), days 2-3, days 6-8, and from 18 healthy controls (Osaka cohort 2). The plasma resistin, inflammatory cytokines (IL-6, IL-8, MCP-1 and IL-10) and endothelial cell adhesion molecules (ICAM-1 and VCAM-1) were compared between patients and control. Correlations among resistin, inflammatory cytokines and endothelial cell adhesion molecules were evaluated in COVID-19 and sepsis.

**Results:**

In the MGH cohort, the day 1 resistin levels were associated with disease severity score. The non-survivors showed significantly greater resistin levels than survivors on days 1, 4 and 8. In the Osaka cohort 1, 28-day non-survivors showed significantly higher resistin levels than 28-day survivors on days 6-8. Patients with late recovery (defined as the day of weaning off mechanical ventilation >12 or death) had significantly higher resistin levels than those with early recovery on day 1 and days 6-8. In the Osaka cohort 2, plasma resistin levels were elevated in COVID-19 and sepsis patients compared to controls at all measurement points and were associated with inflammatory cytokines and endothelial cell adhesion molecules.

**Conclusion:**

Resistin was elevated in COVID-19 patients and was associated with cytokines and endothelial cell adhesion molecules. Higher resistin levels were related to worse outcome.

## Introduction

In 2001, resistin was first discovered in mice as a mediator released from adipocytes and was reported to be associated with obesity and insulin resistance (1). In humans, however, resistin seems to be mainly secreted by macrophages rather than adipocytes (2). Resistin levels are reported to be increased in septic subjects and to be associated with severity and prognosis (3). Hierarchical clustering analysis showed that resistin and inflammatory cytokines formed a network that includes interleukin (IL)-6, IL-8, IL-10 and monocyte chemotactic protein 1 (MCP-1) in the acute phase of sepsis and burns and that this network is associated with severity and prognosis (4, 5). Resistin was also reported to be associated with endothelial cell adhesion molecules, including intercellular adhesion molecule 1 (ICAM-1) and vascular cell adhesion molecule-1 (VCAM-1), in sepsis (6).

Coronavirus disease 2019 (COVID-19) is a new viral disease caused by severe acute respiratory syndrome coronavirus-2 (SARS-CoV-2). COVID-19 was first reported in China (7) in December 2019 and has rapidly spread globally. Although the development of vaccines has reduced the number of patients who become critically ill or die from COVID-19, the number of patients suffering from COVID-19 continues to remain high. As of April 30, 2022, COVID-19 had infected over 510,000,000 people and caused over 6,200,000 deaths (8) worldwide. Although it has been over 2 years since the pandemic began, the pathogenesis of COVID-19 is still not fully understood. Inappropriate host immune response caused by SARS-CoV-2 can lead to excessive inflammation (9–12) called “cytokine storm” (13). Vascular endothelial damage and thrombotic complications leading to acute respiratory distress syndrome (ARDS) and multiple organ dysfunction syndrome have also been reported (14–17). Endothelial cell adhesion molecules such as ICAM-1 and VCAM-1, which were also used as endothelial damage markers, were elevated in COVID-19 and associated with disease severity in previous reports (15, 18).

The role of resistin in COVID-19 has remained unclear. Therefore, this study aimed to evaluate whether resistin is involved in the pathogenesis of COVID-19.

## Materials and Methods

### Cohort Data and Sample Collection

**Figure 1:**
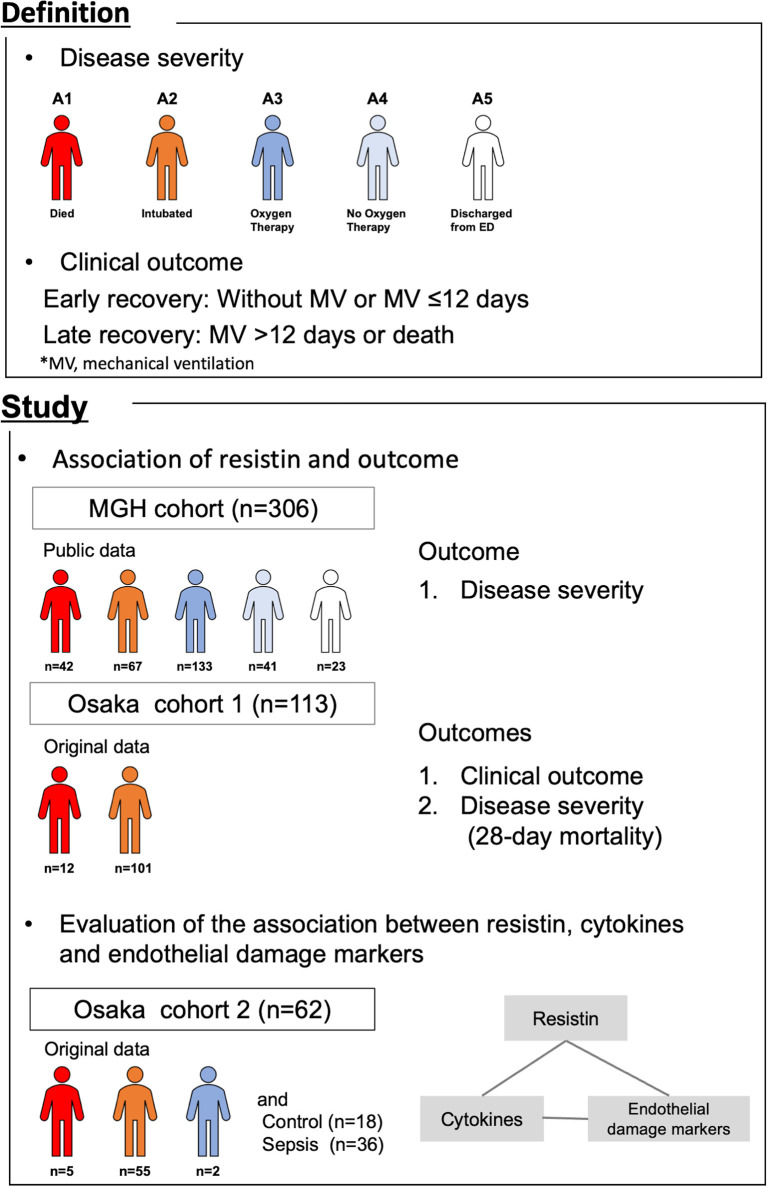
Summary of this study. The association of plasma resistin with outcome was evaluated in the MGH cohort and Osaka cohort 1. The association between resistin, cytokines and endothelial cell adhesion molecules was evaluated in Osaka cohort 2. MGH, Massachusetts General Hospital; MV, mechanical ventilation.

In this study, we used data from three different observational cohorts (**Figure 1**). The “MGH cohort” was comprised of publicly available data provided by the Massachusetts General Hospital Emergency Department COVID-19 Cohort (Filbin, Goldberg, Hacohen) (19) with Olink Proteomics (https://www.olink.com/mgh-covid-study/), which was conducted from March 2020 to April 2020 and included 306 COVID-19 patients. Patient blood samples were collected on days 1, 4 and 8 (maximum of 3 time points/patient). Blood samples on day 1 were considered to be those obtained when the initial clinical blood draw was performed in the Emergency Department. Samples were obtained on days 4 and 8 if the patient remained in hospital. The observational period was from day 1 through day 29. Plasma resistin levels were measured by Olink^®^ Explore 1536. The levels of protein were expressed as the normalized protein expression value (NPX) in log2 scale.

The “Osaka cohort 1” was our original cohort comprising 113 COVID-19 patients admitted to the Department of Traumatology and Acute Critical Care Medicine, Osaka University Graduate School of Medicine and the Osaka Prefectural Nakakawachi Emergency and Critical Care Center from December 2020 to January 2021 and April 2021, who were treated with invasive mechanical ventilation (MV) and whose day 1 plasma samples were obtained. Patient blood samples were collected on days 1 (day of intensive care unit [ICU] admission) and 6-8 (maximum of 2 time points/patient). The observational period was from day 1 through day 28. Plasma resistin levels were measured by ELISA.

The “Osaka cohort 2” was also our original cohort and comprised 62 COVID-19 patients admitted to the same two institutions from August 2020 to December 2020, 36 sepsis patients and 18 healthy controls. Patients with sepsis who were admitted to the Department of Traumatology and Acute Critical Care Medicine, Osaka University Graduate School of Medicine between February 2014 and July 2015 were included as an ICU control group. The patients with sepsis were all over 18 years of age, and all patients met the Sepsis-3 criteria. Patients with COVID-19 and sepsis, blood samples were collected on days 1 (day of ICU admission), 2-3 and 6-8 (maximum of 3 time points/patient) and once from the healthy controls. Plasma resistin, IL-6, IL-8, IL-10, MCP-1, ICAM-1 and VCAM-1 were measured by ELISA.

This study was conducted according to the principles of the Declaration of Helsinki and was approved by the institutional review board of Osaka University Hospital (Numbers: 12007, 16109 and 885 [Osaka University Critical Care Consortium Novel Omix Project; Occonomix Project]). Informed consent was obtained from the patients or their relatives and the healthy volunteers for the collection of all blood samples.

### Definition of Severity and Clinical Outcome

Acuity scores were based on the World Health Organization ordinal outcomes scale (20): A1, dead; A2, intubated, survived; A3, hospitalized with oxygen; A4, hospitalized without oxygen; A5, discharged. Disease severity was classified according to the maximum acuity score during observational period.

COVID-19 patients frequently require prolonged MV due to refractory pneumonia and ARDS. Nearly 30% of COVID-19 patients with MV required tracheostomy due to prolonged MV (21). An observational study evaluating 1890 COVID-19 patients with tracheostomy in Spain revealed that the median day of tracheostomy was 12 days after intubation and that 24% of these patients remained on MV support after one month (22). Prolonged MV management can lead to long-term hospital stays and vast use of ICU resources, thus taking beds away from patients with other diseases that usually require ICU management. In fact, increased mortality from other diseases has been reported during the COVID-19 pandemic (23, 24). In this study, we defined the clinical outcome of patients who were treated with MV for ≤12 days as early recovery and >12 days and death as late recovery as in our previous study (25).

### ELISA Assay

Plasma samples were stored at -30°C until use. ELISAs (R&D Systems, Minneapolis, MN, USA) were performed to measure the plasma levels of resistin, IL-6, IL-8, IL-10, MCP-1, ICAM-1 and VCAM-1. After thawing of frozen plasma samples, measurement was conducted according to the manufacturer’s protocol. A microplate reader (SH-9000Lab; Corona Electric Co., Ltd., Ibaraki, Japan) was used to measure absorbance. The minimum detectable levels were as follows: resistin, IL-8, IL-10 and ICAM-1: 31.2 pg/mL; MCP-1 and VCAM-1: 15.6 pg/mL; and IL-6: 9.4 pg/mL.2.4.

### mRNA Expression of Resistin

Ten patients with COVID-19 and 5 healthy controls in the Osaka cohort 2 were selected randomly, and total RNA isolation of leukocytes from patients on day 1 and healthy controls was performed using a PAXgene™ Blood RNA System (BD Bioscience, San Jose, CA, USA). The collection tubes were stored after blood collection until further analysis at -30°C. Library preparation was performed using a TruSeq stranded mRNA sample prep kit (Illumina, San Diego, CA, USA) in accordance with the manufacturer’s instructions. Sequencing was performed on an Illumina NovaSeq 6000 platform in 101-base paired-end mode. The sequenced reads were mapped to the human reference genome sequences (hg19) using TopHat, version 2.0.13, in combination with Bowtie2, version 2.2.3, and SAMtools, version 0.1.19. The fragments per kilobase of exon per million mapped fragments were calculated using Cufflinks, version 2.2.1. The gene-level expression raw read counts were calculated using featureCounts. The raw data from this study were submitted under Gene Expression Omnibus accession number GSE192707 for future access.

### Statistical Analysis

Values are reported as n (%) and the median value (quartile 1–3) if the data distribution was skewed, or as the mean ± SD unless stated otherwise.

In the MGH COVID-19 cohort, the patients were divided into four groups based on the quartiles of the day 1 resistin NPX. The proportion of disease severity was calculated for each group. The difference in the proportion was compared using the chi-squared test. The Wilcoxon rank-sum test was used to evaluate the differences between survivors (A2-A5) and non-survivors (A1) on days 1, 4 and 8.

In Osaka cohort 1, the patients were divided into two groups, the early recovery group and late recovery group or 28-day survivors and 28-day non-survivors. The plasma resistin levels were compared between two groups by Wilcoxon rank sum test.

In Osaka cohort 2, resistin, inflammatory cytokines and endothelial cell adhesion molecules were transformed to logarithmic values to normalize the data distribution before the analyses. Dunnett’s test was used to evaluate the difference of each value between patients and healthy controls. Correlations between resistin, inflammatory cytokines and endothelial damage markers were evaluated by Spearman correlation coefficients. Correlations were visualized by Cytoscape^®^ software (www.cytoscape.org) version 3.8.0. Log2 fold changes were calculated by dividing the average mediator levels in COVID-19 and sepsis by the average levels in healthy controls. The patients were divided into two groups: 28-day survivors and 28-day non-survivors or early recovery and late recovery in COVID-19. Wilcoxon rank-sum tests were used to evaluate differences between two groups on each day. The resistin levels were compared between day 1 and day 2-3, day 1 and day 6-8 or day 1 and day 6-8 by Wilcoxon signed-rank test for each group (early or late recovery).

The mRNA expression of resistin was compared between COVID-19 patients and healthy controls by a Wilcoxon rank-sum test.

The association between day 1 severity of disseminated intravascular coagulation and the levels of resistin was assessed by the International Society of Thrombosis and Haemostasis (ISTH) overt DIC score (26). The associations between day 1 resistin levels and platelet counts, D-dimer, fibrinogen and PT (INR) were evaluated by Spearman correlation coefficients.

Statistical analyses were performed using the R software program (version 4.0.2; R Foundation for Statistical Computing, Vienna, Austria). Data are presented using the GraphPad Prism software program (version 8.4.3, GraphPad Software, La Jolla, CA). Statistical significance was defined as P<0.05.

## Results

### Association of Resistin and Disease Severity in MGH Cohort

One of the 306 patient samples in the MGH COVID-19 cohort was identified as an outlier and removed from the final dataset. Accordingly, a total of 305 day 1 samples, 215 day 4 samples and 139 day 8 samples were available in the MGH COVID-19 cohort. In this cohort, 42 patients died (A1) and 263 survived (A2-A5). Sixty-seven of the survivors received MV (**Table 1**).

**Table 1:** Clinical and Demographic Characteristics of COVID-19 Patients in the MGH Cohort and Osaka Cohort 1.

	MGH cohort, Boston, USA (n=306)	Osaka cohort 1, Osaka, Japan (n=113)
Male sex, n (%)	162 (52.9)	80 (70.8)
Age, median years (IQR)	58 (45-75)	65 (55-74)
20–34 years	32 (10.5)	1 (0.8)
35–49 years	66 (21.6)	11 (9.7)
50–64 years	89 (29.1)	44 (38.9)
65–79 years	65 (21.1)	47 (41.6)
Over 80 years	54 (17.6)	10 (8.9)
Comorbidities, n (%)		
Heart disease	48 (16.5)	12 (10.6)
Lung disease	66 (21.1)	12 (10.6)
Kidney disease	41 (14.3)	12 (10.6)
Immunocompromised condition	25 (7.7)	4 (3.5)
Hypertension	146 (48.1)	47 (41.6)
Diabetes	111 (35.4)	41 (36.3)
BMI, median years (IQR)	29 (26-34)	25 (22-28)
0–24.9	46 (15.1)	49 (43.4)
25.0–39.9	205 (66.9)	58 (51.3)
Over 40	35 (11.4)	1 (0.8)
Unknown	20 (6.5)	5 (4.4)
SOFA score, median (IQR)	2 (1-7)	5 (3-6)
Disease severity (Acuity score_max_)		
A1=Died	42 (13.7)	12 (10.7)
A2=Intubated/ventilated, survival	67 (21.9)	101 (85.8)
A3=Hospitalized, O_2_ required, survived	133 (43.5)	0 (0)
A4=Hospitalized, no O_2_ required,survived	41 (13.4)	0 (0)
A5=Discharged	23 (7.5)	0 (0)
Clinical outcome		
Early recovery	–	64 (56.6)
Late recovery	–	49 (43.4)

Data are reported as number (percentage) or median (IQR, interquartile range) as appropriate. BMI, body mass index; Heart disease, coronary artery disease,congestive heart failure, valvular disease; Lung disease, asthma, COPD, requiringhome O_2_ and any chronic lung condition; Kidney disease, chronic kidney disease,baseline creatinine >1.5; Immunocompromised condition, active cancer,chemotherapy, transplant and immunosuppressant agents, asplenic; SOFA,Sequential Organ Failure Assessment.

**Figure 2:**
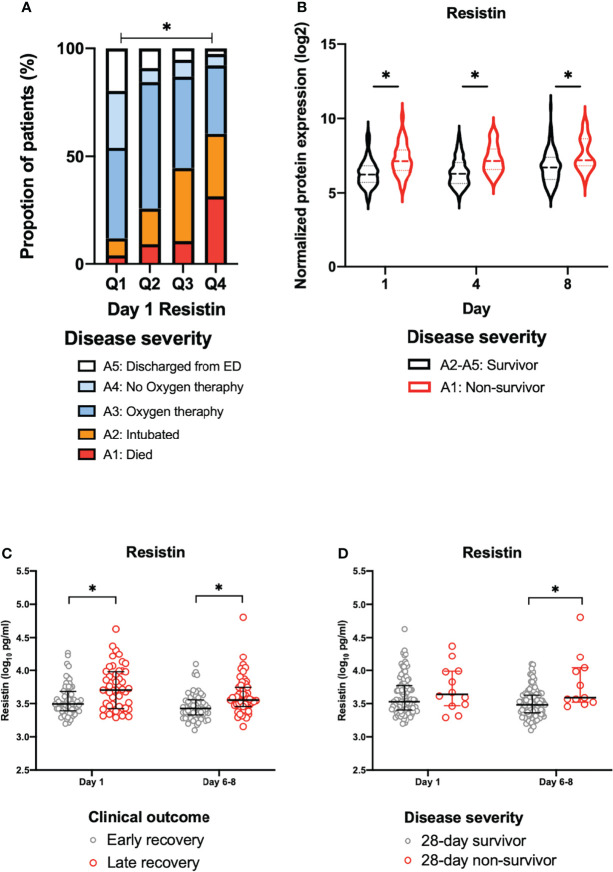
The association between plasma resistin and disease severity or clinical outcome. **(A)** The disease severity by quartiles of day 1 resistin levels in the MGH cohort. Asterisk indicates a statistically significant difference between disease severity and the day 1 resistin levels. **(B)** Change in the normalized protein expression value of resistin in survivors (A2-A5) and non-survivors (A1) on days 1, 4 and 8 in the MGH cohort. Asterisk indicates a statistically significant difference between survivors (A2-A5) and non-survivors (A1) (P < 0.05) on each day. The resistin levels for early recovery and late recovery **(C)** and 28-day survivors and non-survivors **(D)** on each day in Osaka cohort 1. The error bars show the median and upper and lower quartiles. Asterisks indicate a statistically significant difference (P < 0.05) between two groups on each day by Wilcoxon rank sum test. ED, emergency department; MGH, Massachusetts General Hospital.

The day 1 resistin levels were associated with disease severity (**Figure 2A**). In the COVID-19 patients (days 1, 4 and 8), the resistin levels of the non-survivors (A1) were significantly increased in comparison to those of the survivors (A2-A5), as shown in **Figure 2B**.

### Association of Resistin and Clinical Outcome and 28-day Mortality in Osaka Cohort 1

The Osaka cohort 1 included 113 intubated COVID-19 patients (male, n=80; female, n=33). The numbers of blood samples collected on day 1 and days 6-8 were 113 and 110, respectively. Twelve patients (10.7%) were 28-day non-survivors (**Table 1**). The plasma resistin levels in patients with late recovery were significantly higher in comparison to those with early recovery on day 1 and days 6–8 (**Figure 2C**). The plasma resistin levels were statistically higher in 28-day non-survivors than in 28-day survivors on days 6-8 (**Figure 2D**).

### Associations Between Resistin, Cytokines and Endothelial Cell Adhesion Molecules in Osaka Cohort 2

The Osaka cohort 2 included 62 COVID-19 patients (male, n=42; female, n=20), 38 patients with sepsis (male, n=29; female, n=9) and 18 healthy controls (male, n=12; female, n=6). All COVID-19 patients were treated in the ICU, 60 patients (96.8%) received MV, and 5 patients (8.1%) died within 28 days from admission. Sepsis patients were also treated in the ICU: 81.6% were treated with the MV and 26.3% had pneumonia. The median Acute Physiology and Chronic Health Evaluation II score and Sequential Organ Failure Assessment (SOFA) score in the COVID-19 and sepsis patients were 14 and 21, and 5 and 9, respectively. Hospital mortality rates in the COVID-19 and sepsis patients were 8.1% and 23.7%, respectively. The comorbidities and laboratory data are shown in **Table 2**.

**Table 2:** Clinical and Demographic Characteristics of COVID-19 and Sepsis Patients in the Osaka Cohort 2.

	Healthy controls	COVID-19 patients	Sepsis patients
	(n=18)	(n=62)	(n=38)
Age, yrs (IQR)	59 (55-71)	71 (61-76)	76 (65-81)
Sex, male n (%)	12 (66.6)	42 (67.7)	29 (76.3)
BMI, kg/m^2^	21.6 (20.7-25.6)	24.1 (22.6-26.3)	21.6 (19.0-23.5)
Comorbidities, n (%)			
Hypertension	4 (28.5)	33 (53.2)	11 (28.9)
Diabetes	1 (7.1)	27 (43.5)	15 (39.5)
Hyperlipidemia	10 (55.6)	19 (30.6)	7 (18.4)
Laboratory data			
White blood cell (/μL)		7,700 (4,700-14,000)	10,700 (6,800-15,400)
Platelet count (10^4^/μL)		19.8 (15.9-24.0)	12.0 (4.8-26.6)
D-dimer (μg/mL)		2.5 (1.3-4.2)	8.7 (3.8-14.9)
Creatinine (mg/dL)		0.7 (0.5-0.9)	1.6 (0.9-2.3)
Bilirubin (mg/dL)		0.5 (0.4-0.7)	0.7 (0.5-1.3)
CRP (mg/dL)		9.5 (5.3-13.3)	16.0 (7.9-21.6)
Origin, n (%)			
Chest		62 (100)	10 (26.3)
Abdomen		0 (0)	11 (29.0)
Soft tissue		0 (0)	12 (31.6)
Urinary		0 (0)	3 (7.8)
Others		0 (0)	2 (5.3)
APACHE II score		14 (9-17)	21 (14-30)
SOFA score		5 (3-6)	9 (5-13)
MV, n (%)		60 (96.8)	31 (81.6)
Days to weaning off MV		12 (7-55)	9 (3-15)
28- Day mortality, n (%)		5 (8.1)	9 (23.7)

Data are given as the median (25th-75th percentile) or as number (%). APACHE, Acute Physiology and Chronic Health Evaluation; BMI, body mass index; CRP, C-reactiveprotein; IQR, interquartile range; MV, mechanical ventilation; SOFA, Sequential OrganFailure Assessment.

**Figure 3:**
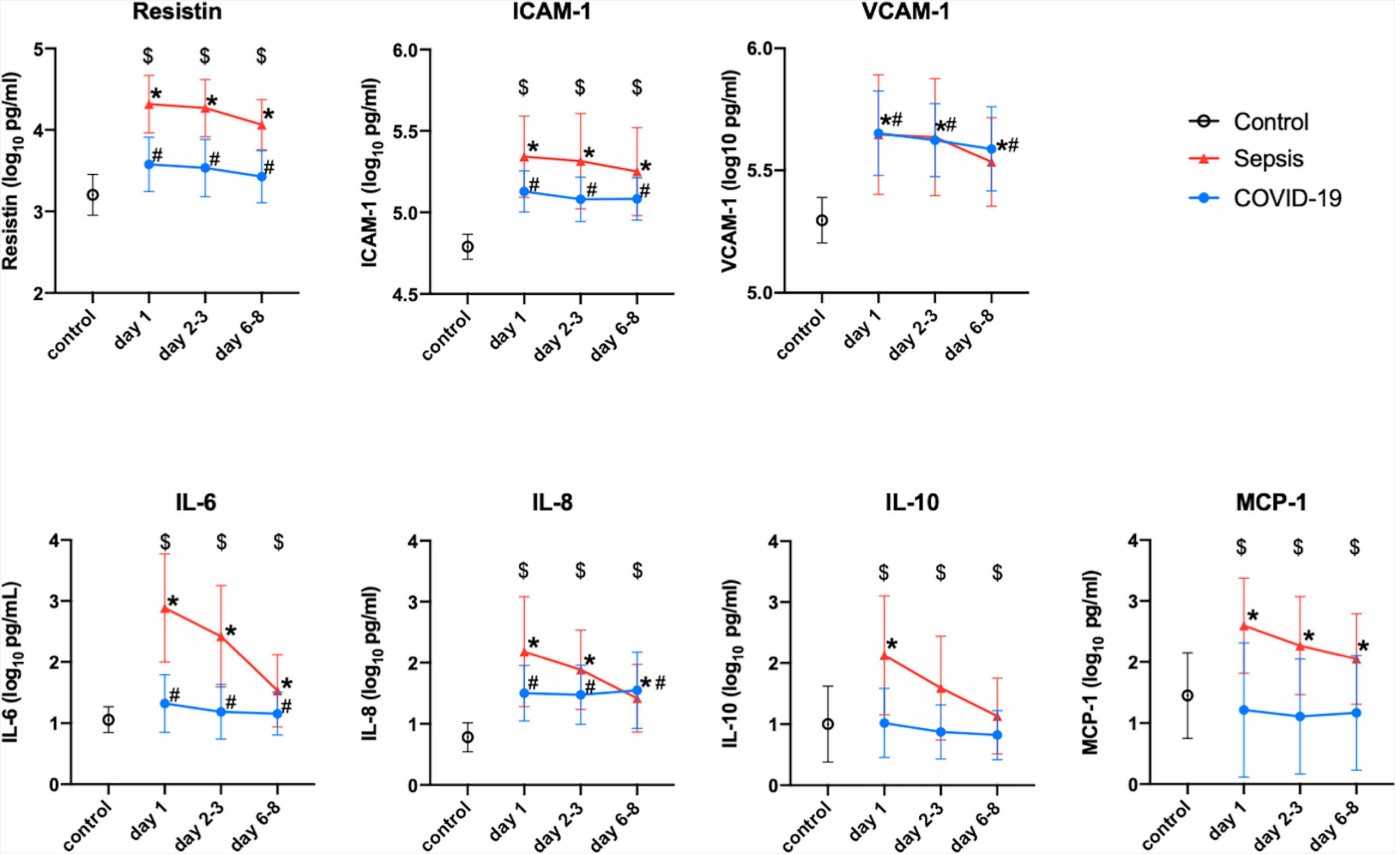
Change in the resistin, ICAM-1, VCAM-1, IL-6, IL-8, IL-10 and MCP-1 levels of Osaka cohort 2. These protein levels were transformed to common logarithmic values to normalize the data distribution. All values are expressed as the mean ± SD. (A) * indicates a significant difference in these proteins between the control and sepsis patients. # indicates a statistically significant difference between the control and COVID-19 patients on each day (*P* < 0.05). $ indicates a statistically significant difference between the sepsis patients and COVID-19 patients. COVID-19, coronavirus disease 2019; ICAM-1, intercellular adhesionmolecule 1; IL, interleukin; MCP-1, monocyte chemotactic protein 1; SD, standard deviation; VCAM-1, vascular cell adhesion molecule-1.

In comparison to those of the healthy controls, the plasma resistin levels of the COVID-19 patients in the Osaka cohort 2 were significantly higher on day 1, days 2-3 and days 6-8. The plasma levels of VCAM-1, ICAM-1 and IL-8 were also higher in the COVID-19 patients than in the healthy control at every measurement point. The plasma levels of resistin, ICAM-1, VCAM-1, IL-6, IL-8 and MCP-1 were all higher than those of the controls. The plasma levels of resistin, ICMA-1, IL-6, IL-10 and MCP-1 of the patients with sepsis were statistically higher than those of the patients with COVID-19 at all measurement points (**Figure 3**).

**Figure 4:**
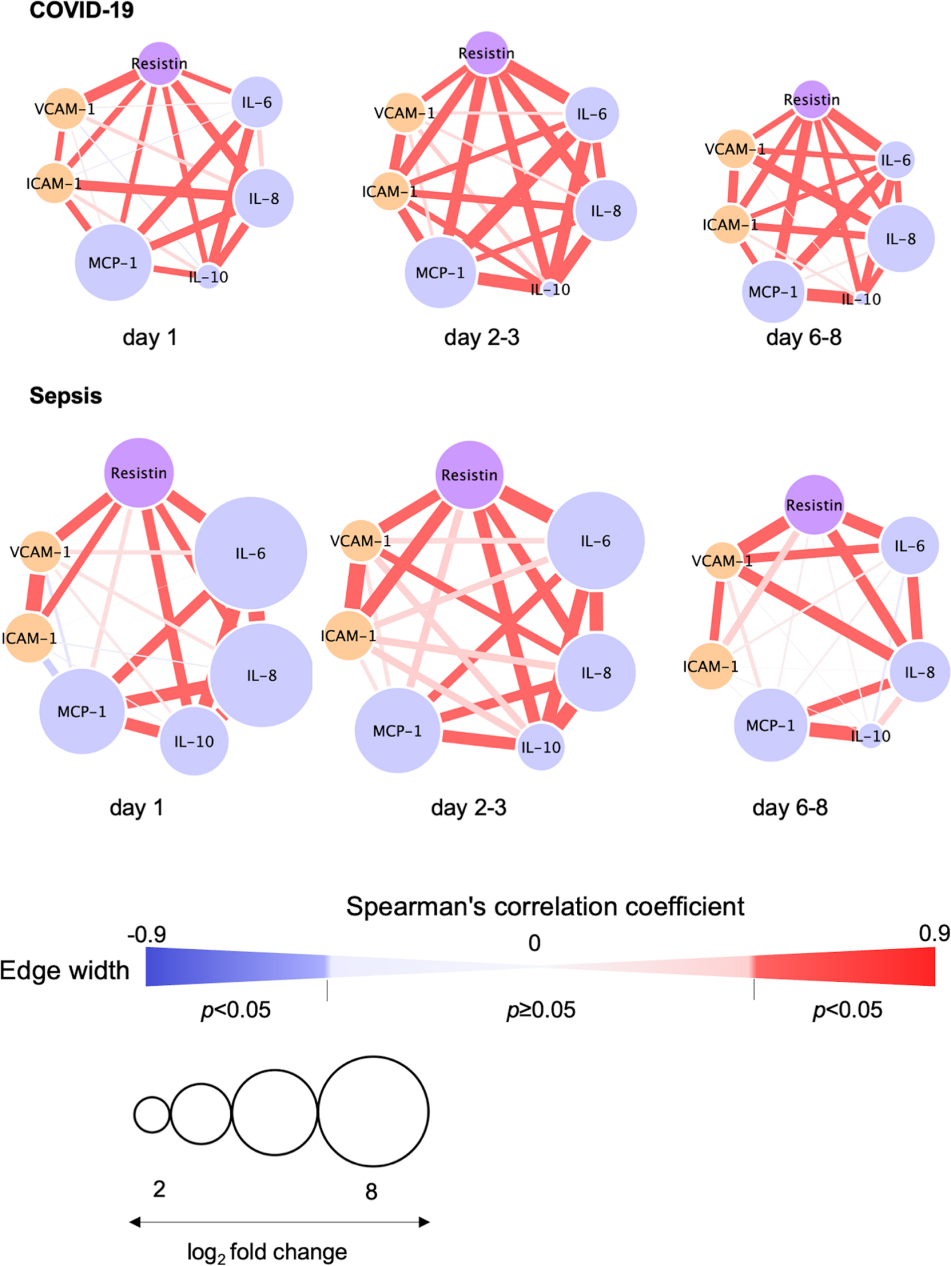
Correlation visualization of resistin, 4 cytokines and 2 endothelial cell adhesion molecules. Resistin, cytokines and endothelial cell adhesion molecules were transformed to common logarithmic values to normalize the data distribution. The width of each edge indicates the Spearman’s correlation coefficients among resistin, cytokines and endothelial cell adhesion molecules. The red edge indicates a statistically significant correlation. The size of each node was determined based on the log2 fold change (i.e., average resistin, cytokine or endothelial damage marker levels in COVID-19 or sepsis patients/average resistin, cytokines or endothelial damage marker levels in controls). Node colors depict resistin (purple), cytokines (light blue) and endothelial damage markers (orange). COVID-19, coronavirus disease 2019; ICAM-1, intercellular adhesion molecule 1; IL, interleukin; MCP, monocyte chemotactic protein; VCAM-1, vascular cell adhesion molecule-1.

The relationships between resistin, IL-6, IL-8, IL-10, VCAM-1 and ICAM-1 on day 1, days 2-3 and days 6-8 are depicted in **Figure 4**. Resistin was significantly associated with IL-6, IL-8, IL-10, VCAM-1 and ICAM-1 in the COVID-19 patients (**Figure 4**).

The plasma resistin levels in patients with late recovery were significantly higher than those with early recovery on days 6–8 (**Supplemental Figure 1A**). There were no differences in the plasma resistin levels of the 28-day survivors and the 28-day non-survivors in the COVID-19 group (**Supplemental Figure 1B**). Decreased plasma resistin levels were observed in the patients in the early recovery group but not in the patients in the late recovery group (**Supplemental Figure 1C**).

### Association Between Coagulopathy and Resistin

The ISTH DIC score was ≥5 in only one patient with COVID-19 and in six patients with sepsis (**Supplemental Figure 2A**). There were statistically significant associations between day 1 resistin and day 1 platelet count, D-dimer, fibrinogen and PT (INR) (**Supplemental Figure 2B**).

### mRNA Expression the Osaka Cohort

The mRNA expression of resistin in whole blood in the COVID-19 patients of the Osaka cohort was significantly higher than that in the controls (**Supplemental Figure 2**).

## Discussion

This is the first study, to our knowledge, to identify an association between resistin, cytokines and endothelial damage markers, and also to identify the relationship between resistin and disease severity and clinical outcome, in COVID-19 patients.

Resistin is reported to play a role as a pro-inflammatory cytokine (27) and to be related to the pathogeneses of cardiovascular disease (28), cancer (29) and sepsis (3, 5, 30). We evaluated 8 cytokines and 8 adipocytokines in both sepsis and burns and concluded that resistin forms a network with IL-6, IL-8, IL-10 and MCP-1 by hierarchical clustering analysis based on Spearman correlation. Resistin was also associated with the SOFA score (31) and the ISTH DIC score in patients with sepsis and burn. Resistin and these four cytokines were measured in the present study, and COVID-19 patients showed increased plasma resistin levels and an association with these cytokines. The cytokine levels in sepsis were reported to be noticeably higher than those in COVID-19 (32). In the present study, the resistin level in sepsis, as well as those of the cytokines, was higher than that in COVID-19. This data shows that the systemic immune reaction in COVID-19 was not specific but common and that the immune reaction and coagulopathy were mild in COVID-19 compared to those in sepsis. Several mechanisms of resistin secretion in sepsis have been reported (33, 34), but the mechanism in COVID-19 has remained unclear. In humans, resistin is delivered from peripheral blood mononuclear cells, macrophages and bone marrow rather than from adipocytes (2, 35, 36). In the present study, resistin gene expression in whole blood cells was elevated, suggesting that these cells were responsible for the production of resistin in COVID-19 patients. *In vitro*, resistin has been shown to induce the nuclear translocation of NF-kB transcription factors in macrophages and to lead to the increased expression of several pro-inflammatory cytokines, including IL-1, IL-6, IL-12 and TNF-α, in both mice and humans (37). In contrast, a few reports showed that inflammatory cytokines such as IL-6 promoted resistin expression *in vitro* (38). Resistin and inflammatory cytokines could be stimulated by each other, thus leading to a cytokine storm in COVID-19.

Endothelial cells respond to cytokines but can also release cytokines themselves and cause inflammation represented as crosstalk between coagulation and inflammation. Furthermore, endothelial cells can express adhesion molecules and growth factors that may promote the inflammatory response in sepsis (39). Cytokine-driven endothelial damage was reported to be an important factor in the pathogenesis COVID-19 (40). In the present study, we showed that ICAM-1 and VCAM-1 were elevated more in COVID-19 patients than in controls and were associated with resistin levels. Resistin was reported to induce mRNA expression of adhesion molecules through NF-kB in vascular endothelial cells (41). Resistin was also reported to stimulate adhesion of monocytes to vascular endothelial cells and induces aggravation of an inflammatory condition in the vessel walls (42, 43). Resistin might thus be involved in the endothelial damage observed in COVID-19.

The relationship between cytokines and endothelial damage is well known, and in this study, we showed that resistin was also strongly associated with cytokines and endothelial cell adhesion molecules as well as being associated with disease severity and clinical outcome in two cohorts. These findings suggest that the systemic inflammation and endothelial damage reported in COVID-19 (13-15) might be caused by resistin and contribute to the pathogenesis of COVID-19.

The present study has several limitations. First, the Osaka cohort only included COVID-19 patients treated in the ICU. Consequently, this cohort did not represent the entire COVID-19 population. Second, the measuring points are based on the time from admission, and thus, the time from onset was not considered. Third, unmeasured confounders such as treatment details are lacking that might have biased the results. Finally, the number of patients in Osaka cohort 2 was relatively small to detect differences in mortality.

## Conclusion

Resistin was elevated in COVID-19 and was associated with both cytokines and endothelial cell adhesion molecules. Higher resistin levels were related to a worse clinical course in patients with COVID-19.

## Data Availability Statement

The datasets presented in this study can be found in online　repositories. The names of the repository/repositories and accession number(s) can be found below: NCBI GEO, accession no: GSE192707.

## Ethics Statement

The studies involving human participants were reviewed and approved by Osaka University Hospital. The patients/participants provided their written informed consent to participate in this study.

## Author Contributions

TE conceived and designed this study, acquired data, analyzed and wrote the manuscript. HisM helped with designing the study and data interpretation and conducted the literature review. TM, YT, TK, HirM, HH, and HY contributed to data acquisition. FS and DO helped analyze the data. SN helped with designing the study. HO conducted the literature review. All authors have read and understood journal’s policies and believe that neither the manuscript nor the study violates any of these. All authors meet the authorship criteria detailed in the submission guidelines, and all authors agree with the contents of the manuscript. All authors contributed to the article and approved the submitted version.

## Funding

This study was supported by JSPS KAKENHI Grant Number 20K17892 and Japan Agency for Medical Research and Development Grant Number 20fk0108404h0001.

## Conflict of Interest

The authors declare that the research was conducted in the absence of any commercial or financial relationships that could be construed as a potential conflict of interest.

## Publisher’s Note

All claims expressed in this article are solely those of the authors and do not necessarily represent those of their affiliated organizations, or those of the publisher, the editors and the reviewers. Any product that may be evaluated in this article, or claim that may be made by its manufacturer, is not guaranteed or endorsed by the publisher.

## Abbreviations

APACHE, Acute Physiology and Chronic Health Evaluation; AUC, area under the curve; BMI, body mass index; CI, confidence interval; COVID-19, Coronavirus disease 2019; ICAM-1, intercellular adhesion molecule 1; ICU, intensive care unit; IL, interleukin; MCP-1, monocyte chemotactic protein 1; MGH, Massachusetts General Hospital; MV, mechanical ventilation; NPX, normalized protein expression value; ROC, receiver operating characteristic; SARS-CoV-2, severe acute respiratory syndrome coronavirus-2; SOFA, Sequential Organ Failure Assessment; VCAM-1, vascular cell adhesion molecule-1.
