# Supplementary material for: Resistin Associated With Cytokines and Endothelial Cell Adhesion Molecules Is Related to Worse Outcome in COVID-19
Source: Front Immunol. 2022 Jun 16;13:830061. doi: 10.3389/fimmu.2022.830061 (PMC9243394; doi:10.3389/fimmu.2022.830061)
Supplement: Supplementary file 1 [file DataSheet_1.docx]

Supplemental Material

# Supplemental Data

**Resistin Associated with Cytokines and Endothelial Cell Adhesion Molecules Is Related to Worse Outcome in COVID-19**

**Takeshi Ebihara^1^, Hisatake Matsumoto^1^*, Tsunehiro Matsubara^1^, Yuki Togami^1^, Shunichiro Nakao^1^, Hiroshi Matsuura^1,2^, Shinya Onishi^1^, Takashi Kojima^3^, Fuminori Sugihara^4^, Daisuke Okuzaki^5^, Haruhiko Hirata^6^, Hitoshi Yamamura^2^, and Hiroshi Ogura^1^**

^1^Department of Traumatology and Acute Critical Medicine, Osaka University Graduate School of Medicine, Suita, Osaka, Japan

^2^Osaka Prefectural Nakakawachi Emergency and Critical Care Center, Higashiosaka, Osaka, Japan

^3^Laboratory for Clinical Investigation, Osaka University Hospital, Suita, Osaka, Japan

^4^Core Instrumentation Facility, Immunology Frontier Research Center and Research Institute for Microbial Diseases, Osaka University, Osaka, Japan

^5^Genome Information Research Center, Research Institute for Microbial Diseases, Osaka University, Osaka, Japan

^6^Department of Respiratory Medicine and Clinical Immunology, Osaka University Graduate School of Medicine, Suita, Osaka, Japan

# Supplemental Figures and Tables

## Supplemental Figure 1

The relationship between resistin and clinical outcome or 28-day mortality in Osaka cohort 2. Resistin levels were transformed to common logarithmic values to normalize the data distribution. All data are expressed as the mean ± standard deviation. The resistin levels in the early recovery and late recovery (A) or 28-day survivor and non-survivor (B) groups on each day. * indicates a statistically significant (p<0.05) difference between two groups on each day. (C) Each plot represents a sample, and samples from the same case are connected by a line. * indicates a statistically significant difference (p<0.05) by Wilcoxon signed-rank test.

## Supplemental Figure 2

The number of patients are shown per ISTH DIC score (A). The association between day 1 resistin and PLT, D-dimer, fibrinogen and PT (INR) are shown. Blue circles indicate patients with COVID-19, and red circles indicate patients with sepsis (B). COVID-19 = coronavirus disease 2019, DIC = disseminated intravascular coagulation, ISTH = International Society on Thrombosis and Haemostasis.

## Supplemental Figure 3

The mRNA expression of resistin. Total RNA of whole blood cells was isolated and quantified by RNA-Seq as described in the Methods section. * indicates statistically significant differences between healthy controls and patients with COVID-19. COVID-19 = coronavirus disease 2019, FPKM = fragments per kilobase of exon per million reads mapped.

**
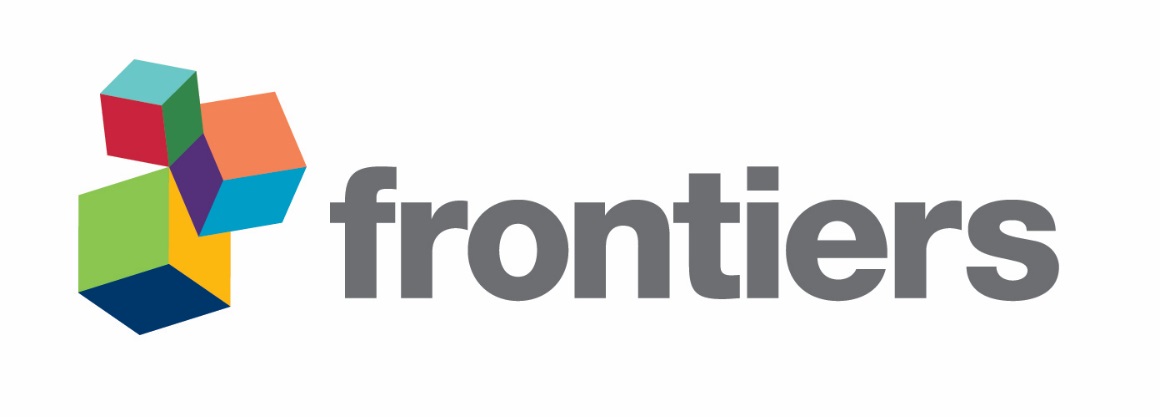
**
